# Motor-effector dependent modulation of sensory-motor processes identified by the multivariate pattern analysis of EEG activity

**DOI:** 10.1038/s41598-023-30324-5

**Published:** 2023-02-23

**Authors:** Kahyun Choi, Sanghum Woo, Joonyeol Lee

**Affiliations:** 1 Center for Neuroscience Imaging Research, Institute for Basic Science (IBS), Suwon, 16419 Republic of Korea; 2 Department of Biomedical Engineering, Sungkyunkwan University, Suwon, 16419 Republic of Korea; 3 Department of Intelligent Precision Healthcare Convergence, Sungkyunkwan University, Suwon, 16419 Republic of Korea

**Keywords:** Smooth pursuit, Saccades, Perception, Motion detection, Human behaviour

## Abstract

Sensory information received through sensory organs is constantly modulated by numerous non-sensory factors. Recent studies have demonstrated that the state of action can modulate sensory representations in cortical areas. Similarly, sensory information can be modulated by the type of action used to report perception; however, systematic investigation of this issue is scarce. In this study, we examined whether sensorimotor processes represented in electroencephalography (EEG) activities vary depending on the type of effector behavior. Nineteen participants performed motion direction discrimination tasks in which visual inputs were the same, and only the effector behaviors for reporting perceived motion directions were different (smooth pursuit, saccadic eye movement, or button press). We used multivariate pattern analysis to compare the EEG activities for identical sensory inputs under different effector behaviors. The EEG activity patterns for the identical sensory stimulus before any motor action varied across the effector behavior conditions, and the choice of motor effectors modulated the neural direction discrimination differently. We suggest that the motor-effector dependent modulation of EEG direction discrimination might be caused by effector-specific motor planning or preparation signals because it did not have functional relevance to behavioral direction discriminability.

## Introduction

Our everyday interactions with the environment consist of receiving sensory information from the sensory organs and taking action. During these sensorimotor processes, the neural representations of identical sensory stimuli may differ when concurring actions differ. Imagine, for example, a pitcher throwing a ball during a baseball game. Although the visual information of the ball that the catcher and referee receive will be almost identical, the intended actions of the two are completely different. Thus, the overall sensory-motor processes might be different from each other, even at the stage of sensory information processing. This study focuses on the differences in sensory-motor processes when effector behaviors differ.

Sensory information processing for identical stimuli can be modulated by different cognitive factors and states, such as attention, prediction, or working memory. Previous studies have shown modulation of neural responses in sensory areas by attention^1–3^ or learning^4^. Other studies have demonstrated the involvement of attention and working memory in multisensory integration processes^5,6^. Modulation of sensory information by the cognitive state is also known to be controlled by frontoparietal attention control regions, such as the frontal eye fields, dorsolateral prefrontal cortex, and intraparietal region^7–9^.

Studies have reported the modulation of sensory information by action states. Human electroencephalography (EEG) studies have demonstrated the locomotion-induced inter-areal synchronization of high-gamma oscillations^10^. A study reported stronger effective connectivity among several brain regions (prefrontal, posterior, and anterior cingulate) in the locomotive state than in the stationary state while participants were performing a cognitive task, suggesting neural modulation by the action state^11^. Animal studies have shown that visual cortical neural responses dramatically increase when mice perform voluntary movements. However, the lateral geniculate nucleus (LGN) response did not change, suggesting that modulation might occur in cortical regions^12,13^. In contrast, strong suppression was observed in the auditory cortex before and during the movement. Therefore, movement-related neural modulation works differently depending on sensory modalities^14,15^. Similarly, different effector behaviors might modulate neural sensory representation. Previous research on monkeys suggests that attentional modulation of neural sensory representation would differ depending on the type of effector behavior used to report the animals’ decisions. One study demonstrates that the attention fields formed in Macaque monkeys’ visual area V4 were more narrowly focused when saccadic eye movement was used as the reporting behavior than when manual response was used as the reporting behavior^16–18^. Therefore, neural sensory representation might be modulated differently by the action state and different types of effector behaviors.

Despite this evidence, most sensorimotor studies have not systematically tested behavior-induced modulation of sensory information^19,20^. Given that either manual responses or saccadic eye movements are frequently used as behavioral reports in perceptual decision-making tasks, it is essential to know if the neural sensory information or decision variables are represented differently when different effector behaviors are employed to report perception. A previous fMRI study observed neural modulation by different behavioral modalities, but they mainly focused on behavior-independent decision variables represented in the frontal cortex^21^. In addition, studies investigating the role of different effector behaviors in sensory information processes in human EEG are scarce. Most EEG researchers avoid using eye movements as behavioral reports because these activities are prone to muscle artifacts^22,23^. Given that saccadic eye movements and smooth pursuit eye movements are natural human behaviors and frequently accompany behaviors for detection, discrimination, and decision-making, it is important to know whether the upcoming oculomotor behaviors or manual response for reporting the perceptual decision have any influence on EEG representation of the sensory input.

In this study, we investigated whether different effector behaviors modulate sensory processes differently when the task and trial structures are similar. We asked human participants to perform a motion direction discrimination task in which the behavioral reports for the perceived motion direction were smooth pursuit eye movement, saccadic eye movement, or button press (manual response). We deliberately designed the experiments such that the sensory motion stimuli used for perceptual decision-making were identical, and only the effector behaviors varied across their conditions. We compared the EEG representations of identical sensory stimuli using a multivariate pattern analysis method.

Using the Mahalanobis distance as a measure of EEG activity pattern dissimilarity, we compared neural representations of the same sensory motion stimulus under different effector behavior conditions. We found that the neural representations differed when the effector behaviors were different, even before any motor actions were performed. Furthermore, the neural sensitivities of the motion direction discriminability during that time were different from each other; it was the most sensitive and fastest when the saccadic eye movement was the effector behavior and the least sensitive and slowest when the button press was the reporting behavior. Despite neural differences, the behavioral performances of reaction time and correct response rate were similar, as shown through a separate behavioral experiment. We suggest that different neural representations for the same sensory input may reflect effector-specific motor planning or preparation signals that are readily captured by EEG recordings.

## Materials and methods

### Participants

EEG and behavioral data were collected from 19 participants (10 females) in the EEG recording experiment, and behavioral data were collected from 10 participants (9 females) in a separate experiment that only recorded behaviors. All the participants had a normal or corrected-to-normal vision. We excluded one participant from the EEG recording experiment because more than 30% of the trials were removed during the EEG data preprocessing. All the participants provided written informed consent prior to each experiment. All study methods followed the relevant guidelines and regulations and were approved in advance by the *Institutional Review Board* of Sungkyunkwan University.

### Stimuli and task design

The experiment was conducted in a quiet and dark room where the only light source was a CRT monitor. Participants were instructed to report the direction of a visual motion stimulus presented on a gamma-corrected CRT monitor (Hewlett Packard P1230, luminance range from 0.78 to 106.03 cd/m^2^, and the luminance of gray background is 55.75 cd/m^2^) with a vertical refresh rate of 85 Hz and a spatial resolution of 1600 × 1200 pixels. The monitor was positioned 57 cm away from the participants. The motion stimulus was a random-dot kinematogram (4.5°-diameter patch with 128 spots, half with white dots and the other with black dots) with 100% coherence and 25% contrast (in the EEG recording experiment), and its mean luminance was the same as the gray background.

**Figure 1:**
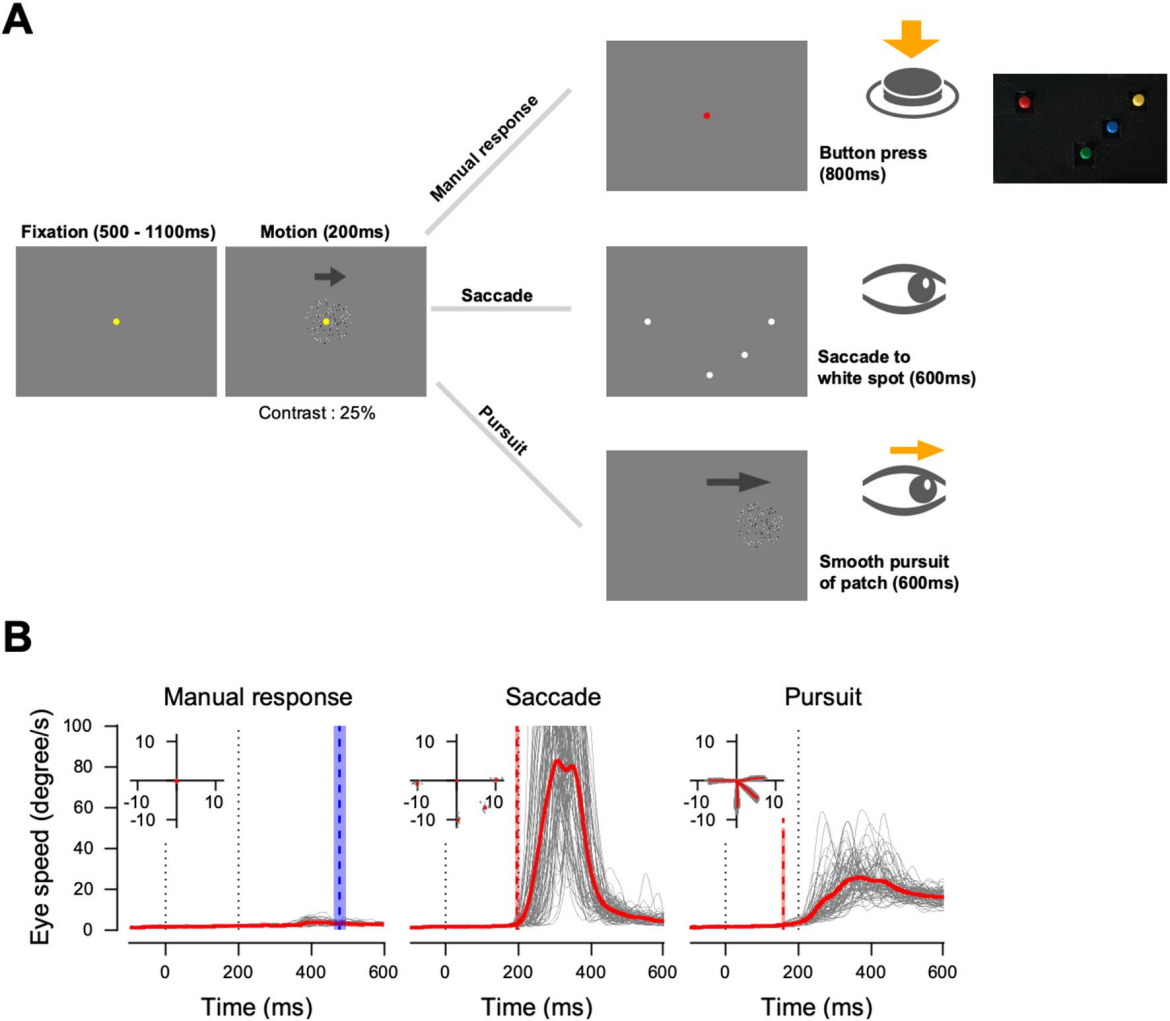
Task design and behavioral results with eye movement traces. (**A**)Task design: Human participants were instructed to report the motion direction of a visual stimulus presented at the center of the screen. During the motion period, only dots inside the patch moved coherently in a specific direction. After this period, participants had to report the perceived direction using button press, saccade, or pursuit eye movements. (**B**) Behavioral results for all participants: Insets show average (red) and individual (gray) eye positions for each direction condition. The main figures show participants’ individual (gray) and average (red) eye speeds. The red dashed lines indicate the average reaction time across participants. The blue dashed line in the left column (manual response) indicates the average reaction time for button presses across participants. Shaded red and blue areas around each dashed line denote standard error. The vertical black dotted lines in each plot indicate the start and end of the motion period.

Participants performed direction discrimination tasks with a manual response (button press), saccade, and pursuit eye movements as behavioral reports. The visual motion stimulus for the decision was identical for all effector behaviors. Figure 1A illustrates the experimental task design. Each trial started when the participants successfully fixated on a yellow dot (0.3°diameter) presented at the center of the screen. It was considered successful if participants did not move their eyes out of an invisible 2° square window centered around the fixation point. After the random fixation period (uniform distribution bounded by 500 and 1100 ms), a random dot patch appeared at the center of the screen. Only dots within the patch moved in one of 4 randomly selected directions (0°, 180°, 270°, and 315°) at a speed of 16 deg/s for 200 ms with 100% coherence. Because most humans are better at tracking visual stimuli with horizontal and downward motion than with vertical and upward motion^24^, we limited the motion directions to the third and fourth quadrants. During the motion period, the yellow fixation dot remained, and participants were instructed to hold their gaze on the dot while paying attention to the direction of the motion generated by the patch of dots. During the motion period, participants were given identical visual stimuli and instructions, regardless of effector behavior conditions. At the end of the motion, the yellow fixation dot was turned off. Participants were then instructed to report the observed direction of motion in one of the following three ways:

1. Manual response condition: A red dot (0.3°diameter) appeared at the center of the screen for 800 ms. During this period, the participants had to fixate on the dot and manually press a button in the response box (see Fig. 1A). Four buttons were located in the response box, and each button had a one-to-one relationship with the corresponding motion direction. Each trial was saved for further analysis if participants maintained fixation within an invisible 2° square around the fixation dot until the end of the trial and pressed one of the buttons within 800 ms. We allowed more time to respond because reaction times in this condition were considerably longer than in other conditions (Fig. 1B).
2. Saccade condition: Four white dots (0.3°diameter) appeared for 600 ms as potential saccade targets at locations 10° away from the center that matched the four directions that the motion of the patch could produce. The participants were instructed to report the perceived direction by shifting their eyes to a dot located in the corresponding location within 600 ms.
3. Pursuit condition: The random dot patch started moving and stayed on for 600 ms in the direction and speed (16°/s) that was the same as the motion. Participants were asked to track the randomly moving patch of dots with their eyes. At the end of 600 ms, the patch stopped moving and stayed on for another 300 ms, which helped the participants complete the smooth pursuit eye movement. If the participants tracked the patch until it disappeared without their eyes leaving the invisible 5° square around the moving stimulus, the trial was deemed successful.

The experiment consisted of blocks with various motor effectors. At the start of each block, participants were informed of which behavioral response was required. The inter-trial interval (ITI) was 800 ms, and each block consisted of 48 trials. To prevent the sequence effect, we shuffled the order of the blocks with different motor effector conditions. To further randomize the experiment, we shuffled six blocks composed of two sets of motor effector blocks (2 × 3). After every six blocks, a minute break was provided to the participants. They performed at least ten blocks per condition, resulting in more than 1440 trials.

In a separate behavioral experiment, we tested whether the use of different motor effectors would affect perception differently (Supplementary Fig. S1A). At 25% of luminance contrast, it was fairly easy for the participants to identify the direction of motion during the motion period. Therefore, we reduced the luminance contrast to 6% to make the task more challenging. In addition, to detect errors in behavioral reports when the effector behavior was smooth pursuit eye movement, we modified the pursuit task as follows: after the motion period, we introduced a 200 ms long blank period. The initiation of pursuit eye movement is guided by the initial sensory motion (typically the first 100 ms from motion onset). Therefore, eye movements during the blank period reflect pursuit initiation induced by the sensory motion stimulus. After the blank period, all four patches appeared and moved radially in four directions (0°, 180°, 270°, and 315°) from the positions where each stimulus started moving from the center of the screen for 200 ms. Four patches continued to move for 400 ms and stopped moving. They remained on for another 300 ms and then disappeared. In other effector behaviors (manual response and saccade task), the task structure was the same as that in the EEG experiment, except for the contrast of the dot patch. Therefore, the modified smooth pursuit eye movement task is similar in structure to other tasks with different effector behaviors. Participants performed at least five blocks per condition, and the total number of trials conducted by each participant exceeded 720.

### Data acquisition

We used an infrared eye tracker (EyeLink 1000 Plus, SR research) to collect the horizontal and vertical eye positions of the participants’ right eyes at a sampling rate of 1 kHz. A real-time data acquisition program (Maestro, https://sites.google.com/a/srscicomp.com/maestro/) was used to control the visual stimulus presentation and the acquisition of eye positions. We collected EEG activity using a 64-channel amplifier (BrainAmp MR plus, Brain Products, GmbH) and active electrodes (actiCAP, Brain Products, GmbH), with a sampling rate of 5 kHz. We kept the impedance of the electrodes under 15 k ${\Omega }$ during the recordings.

We synchronized the EEG data, eye position data, and important events of the experiment using a custom-built Arduino hardware interface. The interface transferred all experiment-related events to the EEG data-collection computer. To ensure accurate timing of the visual stimulus presentation, we used a custom-built photodiode system to store digital events whenever the visual stimulus was turned on.

### EEG preprocessing

We used the following preprocessing pipeline to remove various sources of noise generated during the experiment. The subroutines that we used in the pipeline were part of EEGlab^25^ and FieldTrip^26^ MATLAB toolboxes. First, EEG data were down-sampled from 5 to 1 kHz and high-pass filtered (0.01 Hz) using a Hamming-windowed FIR filter. To remove bad channels, we used the *clean_rawdata* EEGlab plugin, which implements the Artifact Subspace Reconstruction (ASR) routine^27^. We then re-referenced the data using the average EEG activity across channels as a reference and removed line noise (60 and 120 Hz) using the *cleanline* EEGLab plugin. Independent component analysis (ICA)^28^ was performed to remove components related to eye blinks and movements, and ICs with artifactual components were automatically detected using the *ADJUST* EEGlab plugin^29^. None of the participants had a total sum of the explained variance of rejected ICs greater than 50%. Of the total participants, 6.6 ICs, which explained 16.6% of data variance, were rejected on average. After the rejection process, the data were epoched from − 1000 to 2500 ms relative to the trial onset and smoothed using a ± 5 ms boxcar convolution filter to improve the signal-to-noise ratio. Then, baseline correction was performed using the average activity between 400 and 500 ms from the trial onset in each channel and individual trial. Finally, the data were re-epoched from − 300 to 500 ms relative to the motion onset. For most of the analyses, we used EEG activity from 47 out of 64 channels, excluding the ear channels and the most anterior channels, which might be more prone to muscle activity or eye movement artifacts than the others (see the channel topology of Figs. 3B and 4B).

### Behavioral analysis

First, the horizontal and vertical eye positions were smoothed by removing high-frequency signals with a low-pass filter (20 Hz, 2nd order butterworth filter) using the Fieldtrip toolbox. Subsequently, the horizontal and vertical velocity components were obtained using the first derivative of the positions. To further control for the effect of eye movements on EEG activity during the motion period, we excluded trials that contained saccadic eye movements in the time window between − 150 and 150 ms relative to motion onset from further analysis. We then baseline-corrected horizontal and vertical eye velocity data separately using averaged velocities between − 150 and − 50 ms from stimulus onset in individual trials. Reaction time was estimated by comparing the eye speeds after the stimulus onset with the baseline (average eye speed in a time window between − 100 and 0 ms from stimulus onset) at every time point (every millisecond). When the eye speed exceeded five standard deviations from the baseline for 50 ms in a row, the first time was set as the reaction time (see Fig. 1B).

### Multivariate pattern analysis of EEG activity

**Figure 2:**
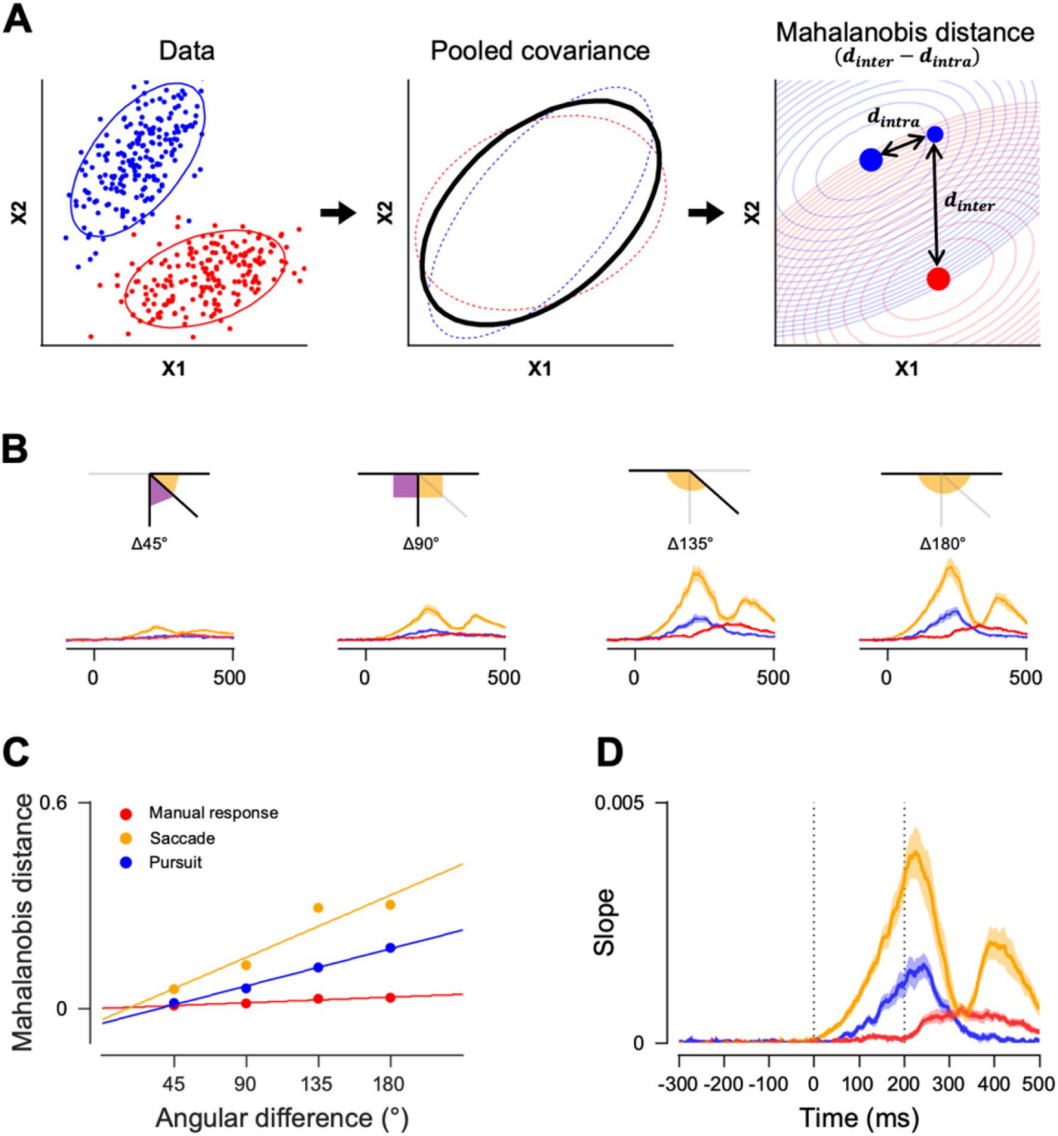
The schematic diagram for Mahalanobis distance calculation and the neural direction discrimination analysis. (**A**) Procedure for calculating dissimilarity between the two multivariate measurements. The inter-group distances were compared with intra-group distances and they were normalized by the pooled covariance. ×1 and ×2 represent two dimensions of example data, shown for illustration purposes. Each contour in the rightmost plot is an equal distance line. (**B**) Mahalanobis distances of the multivariate EEG activity pattern for each angular difference group (45°, 90°, 135°, or 180°), in each effector behavior condition (red: manual response, yellow: saccade, and blue: pursuit). (**C**) The linear regression coefficient of the relationship between the angular differences on the x-axis and corresponding Mahalanobis distances on the y-axis was calculated at each time point. (**D**) Regression slopes are plotted as a function of time for each effector behavior condition. The line and shaded area indicate mean and standard error across participants, respectively.

We estimated the dissimilarity of multivariate EEG activity patterns between two different sensory stimuli or effector behavior conditions using the Mahalanobis distance. As the Mahalanobis distance is obtained by normalizing the Euclidean distance with a covariance matrix, it considers both the signal and noise in the multivariate EEG activity pattern. Therefore, it provides normalized measures for the differences in multivariate EEG activity. Similar to previous studies, we used a leave-one-trial-out procedure to prevent over-fitting (Supplementary Fig. S2)^30^. In each trial, we first excluded the current trial and calculated the centroid of its own group in a multivariate EEG activity pattern. Then we calculated the Mahalanobis distance between the current trial’s activity pattern and the centroid of its own group and the distance between the current trial and the centroid of the comparison group (Fig. 2A). The difference between these two values was used as the dissimilarity score for a given trial. We repeated this calculation across all the trials and averaged them. We also estimated the dissimilarities in behavior by calculating the Mahalanobis distance of eye velocity across different conditions. Because eye velocity is a more sensitive measure of temporal changes than eye position, we only used eye velocity to quantify behavioral dissimilarities.

When we estimated the dissimilarity in eye velocity or EEG activity for different effector behavior conditions, we directly used the dissimilarity estimated from the Mahalanobis distance. However, when neural direction discrimination was estimated, we calculated the ratio between the sensory stimulus differences and neural differences. The Mahalanobis distance of EEG activity patterns was calculated for a given direction pair and compared with the angular differences of the paired physical stimuli. We also estimated the regression line to explain the relationship between them, whereby the slope indicated the sensitivity index for neural direction discrimination. (see Fig. 2B–D). Similarly, behavioral direction discrimination was estimated from eye velocity traces. Mahalanobis distance of eye velocities (horizontal and vertical components) for a stimulus direction pair was compared with the angular differences of the corresponding motion stimulus pair. The slope of the regression line explaining the relationship between the two was used as the sensitivity index for behavioral direction discrimination.

In most of the Mahalanobis distance calculations, EEG data was used from 47 out of 64 channels, excluding data from electrodes prone to muscle artifacts (Fp1, F7, FT9, T7, TP9, TP10, T8, FT10, F8, Fp2, AF7, FT7, TP7, TP8, FT8, AF8, and Iz). When the channels were divided into six subsets along the anterior-to-posterior axis, we used the following overlapping set of channels on each subset (1st subset is the most anterior channels and 6th subset is the most posterior channels):1st subset, AF3, AFz, AF4, F5, F3, F1, Fz, F2, F4, F6, FC5, FC3, FC1, FC2, FC4, and FC6. 2nd subset, F5, F3, F1, Fz, F2, F4, F6, FC5, FC3, FC1, FC2, FC4, FC6, C5, C3, C1, Cz, C2, C4, and C6. 3rd subset, FC5, FC3, FC1, FC2, FC4, FC6, C5, C3, C1, Cz, C2, C4, C6, CP5, CP3, CP1, CPz, CP2, CP4, and CP6. 4th subset, C5, C3, C1, Cz, C2, C4, C6, CP5, CP3, CP1, CPz, CP2, CP4, CP6, P7, P5, P3, P1, Pz, P2, P4, P6, and P8. 5th subset, CP5, CP3, CP1, CPz, CP2, CP4, CP6, P7, P5, P3, P1, Pz, P2, P4, P6, P8, PO7, PO3, Poz, PO4, and PO8. 6th subset, P7, P5, P3, P1, Pz, P2, P4, P6, P8, PO7, PO3, Poz, PO4, PO8, O1, Oz, and O2.

To quantify the relationship between EEG and behavior in direction discriminability, we calculated the cosine similarity between EEG and the behavior for vectors consisting of all direction dissimilarities across every possible stimulus direction pair. The temporal relationship between neural and behavioral direction discriminability was revealed by doing these calculations for different time points. We validated the significance of the relationship using a permutation test. After randomly shuffling the relationship between EEG and behavioral direction discriminability, we calculated the cosine similarity between the two. This procedure was repeated 1000 times to generate a null distribution of cosine similarity. We standardized the original cosine similarity using the mean and standard deviation of the null distribution. To calculate the significance of the results and correct for the multiple comparisons problem, we used a two-sided cluster-based permutation test (*p* < 0.01 for cluster selection and significance test, 10,000 permutations), and a zero value as the baseline for comparison^31^.

### Motion direction expectation analysis

To test if the direction expectation before motion stimulus onset affects EEG direction discrimination, we analyzed the slow drift of eyes during fixation, which would be the behavioral signature of motion direction expectation. Specifically, we tested whether EEG direction discrimination was significantly faster and better when the direction of the eye drifted before the motion onset matched that of the eye velocities after the stimulus motion. We sorted trials by the similarity between spontaneous eye drifts during fixation and pursuit initiation. For this analysis, we used three motion direction conditions (0°, 180°, and 270°) and a support vector machine (SVM) classifier (scikit-learn library) to compare eye movement patterns^32^.

First, we normalized the horizontal and vertical eye velocities separately across conditions based on the time from − 300 to 200 ms relative to the stimulus onset. The SVM classifier was then trained with the eye velocity data from 100 to 200 ms relative to the motion onset, capturing the behavioral reaction to the motion stimulus. To prevent overfitting, we trained a general classifier for each effector using all participants’ data and used a 10-cross validation grid search method. Through this, the optimal hyperparameter set maximizing Matthew’s correlation coefficients was obtained (MCC; we used the same parameters for all three effector behavior conditions: regularization parameter = 100, gamma = 0.001; normalized MCC for the manual response, saccade, and pursuit conditions: 0.71, 0.76, and 0.85, respectively)^33,34^.

Testing data for SVM included the eye drift from − 300 to 0 ms relative to the stimulus onset. To measure the directional eye drift robustly, we first binned the data using a 100 ms overlapping time window, with a 50 ms step size, from − 300 to 0 ms relative to the motion onset and averaged across the time bins. In each trial, the classifier predicted the most probable direction for the test data. We then selected trials that matched evoked eye movements in the current motion direction condition. Finally, through the Mahalanobis distance calculation, we estimated the dissimilarity of EEG activity to two sensory stimuli (0° and 180°) for the direction (of evoked eye movements)-matched or unmatched groups. To ensure that the sample size difference does not affect the Mahalanobis distance calculation, we equalized the number of trials between direction-matched and unmatched groups by randomly selecting the same number of trials (minimum of the two groups) and performed the procedure 10 times (10-iteration of the Monte-Carlo simulation). We then averaged the 10 Mahalanobis distances for each condition.

## Results

Participants performed a direction discrimination task while recording EEG and eye movement data (19 participants). They were asked to report the perceived motion direction using three different behavioral responses (button press, saccade, and pursuit); however, the sensory motion stimuli used for direction discrimination were identical across the reporting conditions. We used Mahalanobis distance as the primary measure for multivariate dissimilarity analyses. Whereas the Euclidean distance simply calculates the pattern differences, the Mahalanobis distance uses the covariance structure of the data to normalize the Euclidean distance, thereby quantifying pattern dissimilarity considering the reliability $\left( {\frac{1}{{\sigma^{2} }}} \right)$ of the data^35,36^.

### Effector-dependent modulation of EEG responses to the identical sensory stimulus

**Figure 3:**
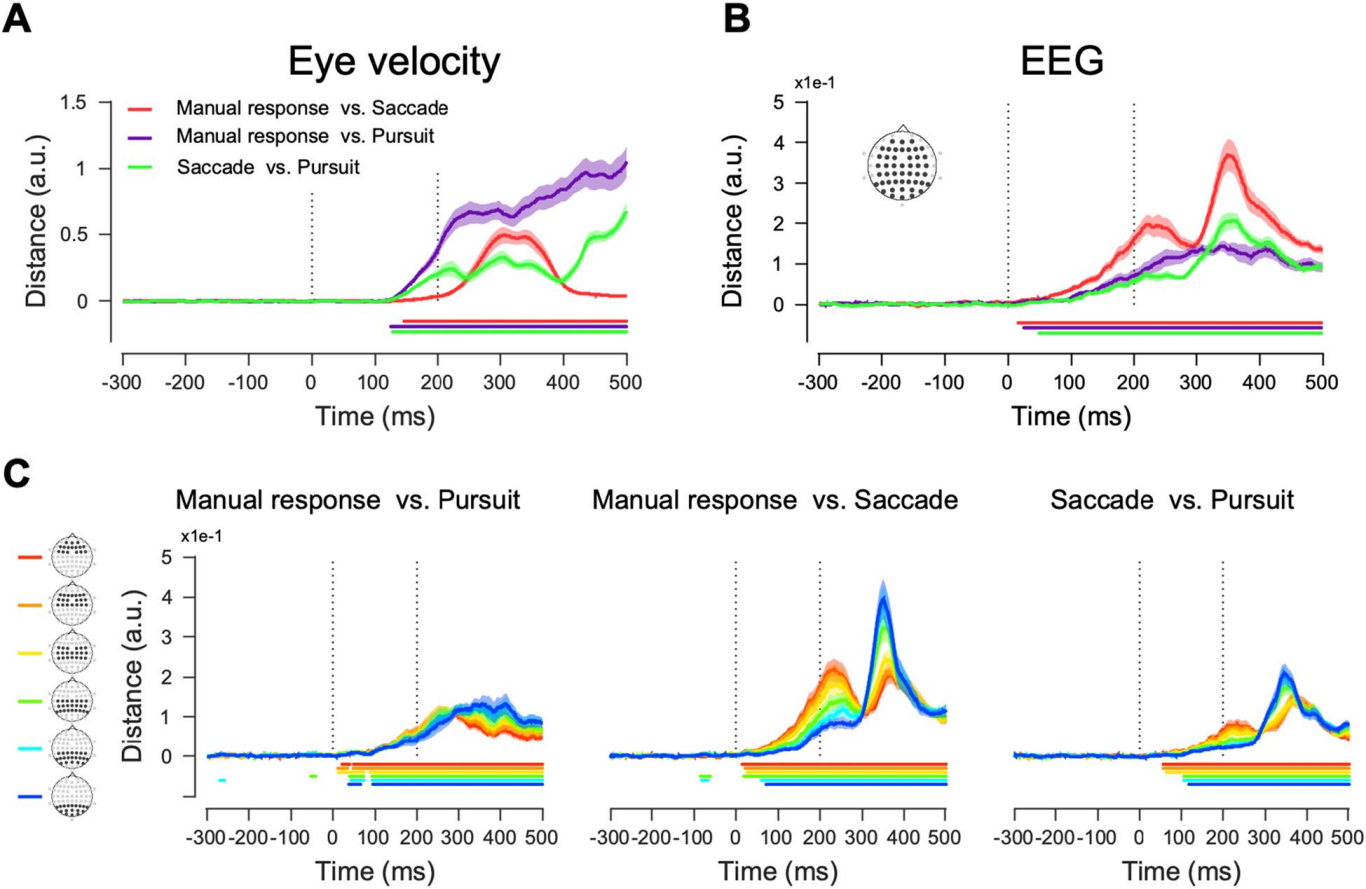
Mahalanobis distances between eye velocities and multivariate EEG activity patterns comparing different effector behavior conditions when a given stimulus’s motion direction is the same. (**A**) Eye velocity dissimilarities showing pairwise comparisons among motor effector conditions using manual response, saccade, and pursuit. (**B**) EEG dissimilarities showing pairwise comparisons among the three motor effector conditions. The recorded data from 47 electrodes were used for calculating the Mahalanobis distances. (**C**) EEG dissimilarities were estimated from six subsets of channels. Means and standard errors across participants were plotted as colored solid lines and shaded areas, respectively. Vertical dotted lines indicate the motion period, and color lines under the plot show the time points where the distances are significantly different from the baseline (two-sided cluster-based permutation test, *p* < 0.01).

We first compared eye movements across different effector behavior conditions (Fig. 3 and Supplementary Fig. S4A). As expected, eye velocities in the pursuit condition differed significantly from those in the manual response or saccade conditions from 125 ms after the target motion onset (Fig. 3A, purple and green, respectively). These temporal dynamics of eye movement dissimilarity were not surprising, given that the nominal response latency of smooth pursuit initiation was about 100 ms^37,38^. In our case, it was about 158 ms on average because we used a pursuit target with lower contrast motion stimulus (25%) than usual (Supplementary Fig. S1B). Eye movement dissimilarity between the saccade and manual response conditions started being significant at 146 ms from stimulus motion onset and became robust after 200 ms. This pattern is also consistent with the average saccade latency of 196 ms in our samples (see Fig. 1B and Supplementary Fig. S1B). These analyses demonstrate that eye movements were quantitatively the same up to 125 ms after the onset of sensory motion, regardless of effector behavior conditions.

As eye movements and visual inputs were the same, EEG responses before 125 ms from motion onset should be identical if the sensory information processes are the same across the effector behavior conditions. However, our results show that this was not the case (Fig. 3B). EEG response pattern comparison between the saccade and manual response conditions revealed significant dissimilarity from 16 ms after the onset of sensory motion (see Fig. 3B, red color). The neural dissimilarity in the saccade-manual response pair contrasted with the behavioral dissimilarity; the appearance of the neural dissimilarity was the fastest, while the appearance of the behavioral dissimilarity was the slowest. This result suggests that there might be effector-specific modulations in neural sensory responses.

To understand which brain regions had the maximum effect, we divided the channels into six subsets, extending from the anterior to posterior regions. Figure 3C shows the channel-specific EEG pattern dissimilarities for the combinations of pairs. In all three pairs, the anterior regions showed the fastest and strongest neural dissimilarity, suggesting that the EEG activity pattern differences could be due to oculomotor-specific motor preparation or movement planning. Surprisingly, the manual-oculomotor behavior pairs (manual response-pursuit and manual response-saccade) showed a significant EEG pattern difference even in the posterior channels (cyan and blue colors in Fig. 3C), before the significant eye movement difference (the fastest significant eye movement difference was at 125 ms after motion onset in manual response-pursuit pair, Fig. 3A, violet color).

In contrast, in the posterior channels, the EEG pattern difference appeared after the significant eye movement difference in the saccade-pursuit pair. EEG representations in the posterior channels (possibly reflecting sensory responses) before any significant motor action were similar to each other (significant EEG dissimilarity in the saccade-pursuit pair appeared at 119 ms from stimulus onset in posterior channels, Fig. 3C, right column plot blue color). However, when the effector behavior was a manual response, the EEG representations in the posterior channels were different from those when the effector behaviors were oculomotor actions in early responses (significant differences appeared at 38 ms from stimulus onset in the manual response-pursuit pair, 72 ms in the manual response-saccade pair, Fig. 3C, left and middle column plots blue color). This suggests that oculomotor planning or preparation signals appear to modify the sensory motion information represented in the visual cortical areas.

### Effector-dependent modulation of EEG direction discrimination

**Figure 4:**
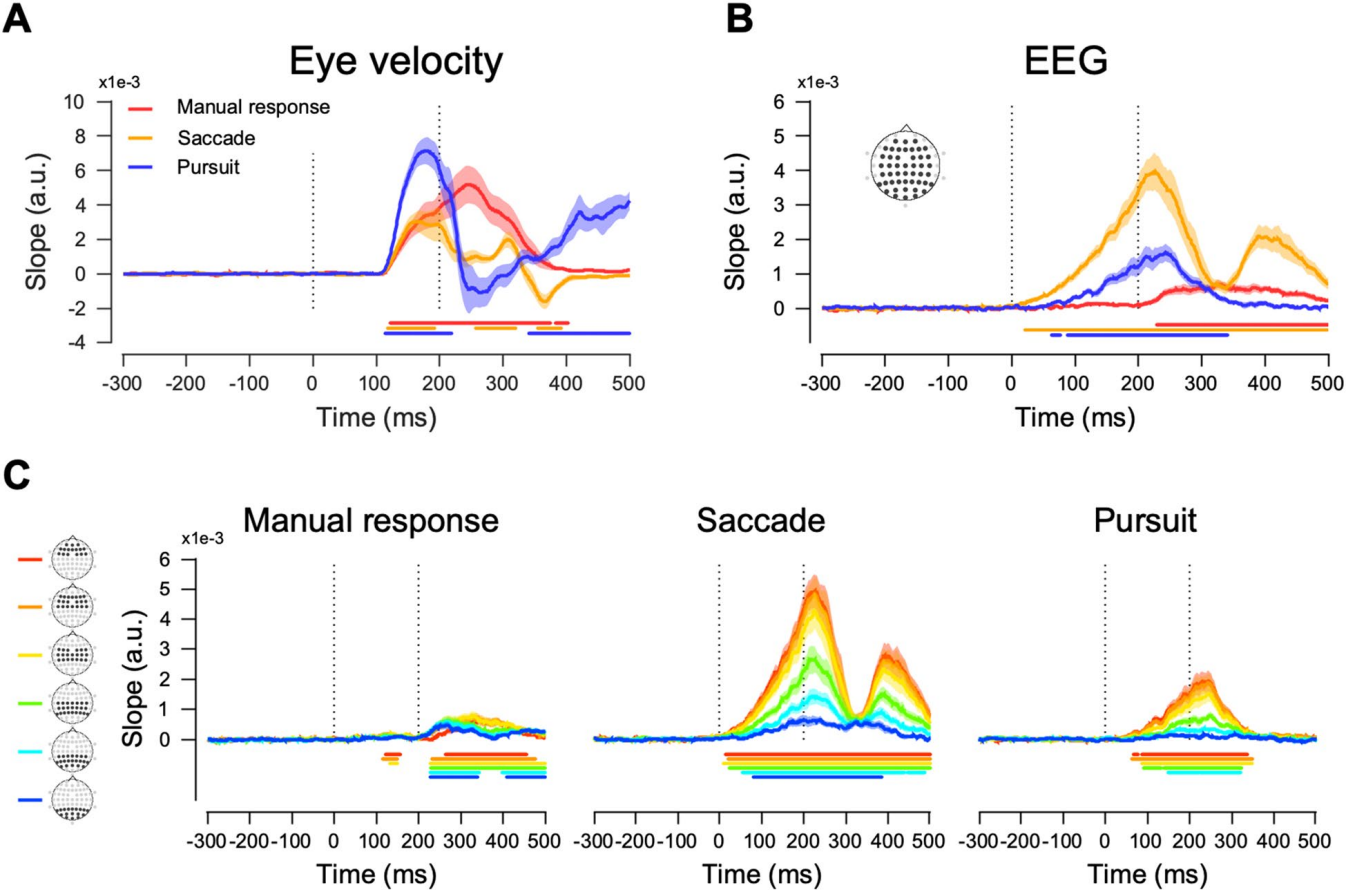
Behavioral and neural direction discrimination. Indices for direction discrimination were estimated from the linear regression analysis for (**A**) Eye velocity, (**B**) EEG data, and (**C**) EEG data divided into six groups of channels. The motion period was marked with vertical dotted lines, and shaded regions denote the standard errors. Colored lines under each plot denote the time clusters that were significantly different from the baseline (two-sided cluster-based permutation test, *p* < 0.01).

Next, we investigated whether neural sensitivities to sensory motion direction changes would be modulated by the choice of motor actions. In a previous study, it was shown that neural direction discrimination of human participants conducting a smooth pursuit eye movement task could be readily quantified by multivariate analysis of EEG activity^39^. Similarly, we estimated the motion direction discrimination in eye movements and EEG activity patterns using the Mahalanobis distance. We ran a linear regression between angular differences (45°, 90°, 135°, or 180°) in the stimulus motion direction and the corresponding Mahalanobis distances for EEG activity, eye velocity, and eye position (Supplementary Figs. S3 and ﻿S4B). Then, we estimated a linear relationship between the two (eye velocity and EEG activity, or eye position and EEG activity) and used the regression slope as an index of direction discrimination, which showed neural and behavioral sensitivities to motion direction changes. The regression slopes for eye velocity were significantly higher than zero from 114 ms after motion onset for all three motor-effector conditions (Fig. 4A, 114 ms, 118 ms, and 122 ms for pursuit, saccade, and manual responses, respectively), indicating that eye movements can discriminate different stimulus motion directions reliably after 114 ms relative to motion onset. In the smooth pursuit eye movement condition, this was not surprising given that the nominal pursuit latency was approximately 100 ms (in our case, it was 158 ms on average because of the lower luminance contrast of the target). However, significant regression slopes in the manual response and saccade conditions that appeared 118 ms after motion onset were unexpected. The average saccade latency was 196 ms (Fig. 1B), and there was no reason for deliberate eye movements when the button press was the motor action. These significant directional components could occur because eye drifts respond to motion stimuli (ocular following), which is difficult to prevent.

Neural direction discrimination showed completely different temporal dynamics under different effector behavior conditions (Fig. 4B). This occurred before any eye movements in the saccade and smooth pursuit eye movement conditions and 229 ms after the target motion onset in the manual response condition. The strength of neural discrimination was the largest in the saccade condition and the smallest in the manual response condition. This result was surprising because the regression slopes measured how much the EEG activity pattern difference discriminated against the motion direction difference. Prior to any motor action differences (~ 125 ms from stimulus onset), the sensitivity of EEG responses to sensory motion direction changes was significantly modulated by what motor action was going to be taken. To further observe which brain regions contributed to elevated or diminished neural sensitivity to sensory motion direction changes, we divided the electrodes into six groups again and obtained regression slopes in each group of electrodes (Fig. 4C). Similar to the previous analysis, neural direction discrimination was fastest and most potent in anterior channels and slowest and weakest in posterior channels, further suggesting that the difference in neural direction discrimination probably originated from motor preparation or planning signals measured in anterior electrodes.

**Figure 5:**
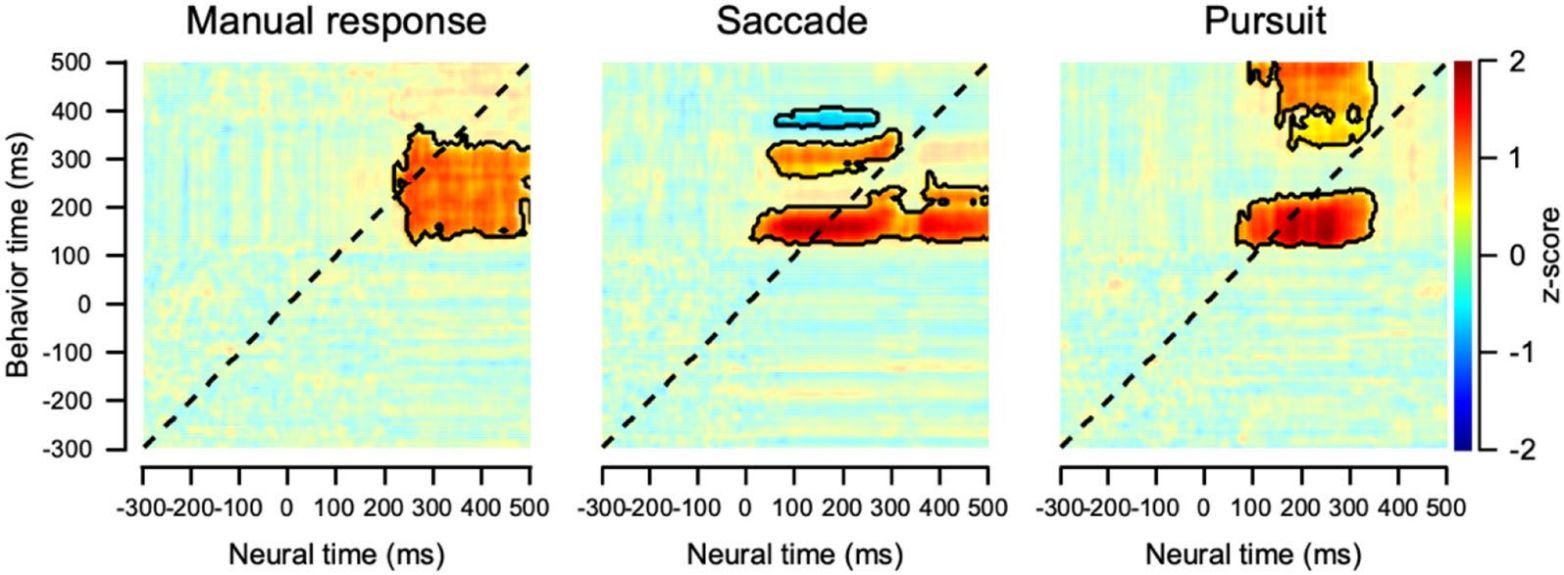
Cross-temporal correlation between neural and behavior direction discriminations. Clusters with significant correlations (that were significantly larger than the baseline) were marked with black contour lines (two-sided cluster-based permutation test, *p* < 0.01). The dotted diagonal indicates correlations between EEG and eye velocities obtained concurrently. Therefore, the significant correlation in the upper-left of the diagonal shows the neural direction discriminability preceding the behavioral direction discriminability. The lower-right area of the diagonal shows the opposite.

Although EEG direction discrimination is generally quicker than behavioral (oculomotor) direction discrimination, this temporal relationship does not guarantee that EEG signals guide eye movements. It is possible that EEG and behavioral direction discrimination are independent of each other. To quantitatively confirm the temporal relationship^40^, we calculated the cross-temporal correlation between EEG and eye velocity vectors constructed from dissimilarity for all direction condition pairs (Fig. 5, see “Methods” for details). By calculating cosine similarities between EEG direction and behavioral direction discriminability over time, we were able to determine whether EEG discrimination significantly influenced behavioral discrimination or vice versa. In the saccade and pursuit conditions, a significant correlation appeared mainly at the upper left off-diagonal of the correlation matrix, suggesting that the neural dissimilarity pattern influences behavioral direction discriminability. However, in the manual response, a significant correlation is observed at the lower right off-diagonal of the matrix, suggesting that the direction discrimination estimated from involuntary eye drift affects the EEG direction discrimination that appeared later. Therefore, the EEG direction discrimination observed in the manual response might have originated from involuntary eye drifts. It should be noted that EEG direction discrimination still occurred before the manual response (button press, Fig. 1B; average reaction time was 478 ms). Therefore, they could still contribute to perceptual direction discrimination that occurred later and reflected the perceptual decision-making process.

## Discussion

In this study, we compared EEG responses to identical sensory stimuli under different effector behavioral conditions using the multivariate pattern analysis method. In addition, we compared the neural direction discrimination of identical sensory stimuli under different effector behavioral conditions. We found that they were different from each other in the initial sensory responses, even before any significant behavioral differences appeared. Moreover, neural direction discrimination was best when saccadic eye movement was used as the motor behavior, and it was worst when manual response was used as the reporting behavior. These results suggest that the EEG representation of a sensory stimulus could vary depending on the type of motor action used to report the perceived sensory stimulus.

### Modulation of neural sensory representation by the selection of the effector behavior

Previous studies investigating decision-making suggested that the threshold crossing of accumulated noisy sensory evidence triggered behavioral responses^20,41^. The rate of evidence accumulation for a specific decision is closely related to the confidence level of perception, which might reflect the strength of sensory information. Therefore, the stronger neural direction discrimination observed in saccadic eye movements in our results could be attributed to stronger sensory representation.

To test this possibility, we conducted an additional behavioral experiment on 10 participants. To compare their performance level, we increased the task difficulty by reducing the luminance contrast of the sensory stimuli from 25 to 6% (Supplementary Fig. S1C and D). We modified the smooth pursuit eye movement task so that the overall task structure across different effector behavior conditions would be comparable (see Methods section for details). Participants demonstrated similar behavioral performance regardless of effector behaviors (71.5 ± 6.5%, 73.1 ± 7.8%, 70.3 ± 7.3% for the manual response, saccade, and pursuit, respectively; see Supplementary Fig. S1D). Therefore, stronger and faster neural direction discrimination in saccadic eye movements did not appear to have any functional meaning—it did not lead to better direction discrimination in behavioral responses.

Why would the EEG representation for identical sensory stimulus differ depending on the choice of the motor effector? As suggested, this could be explained by differences in neural signals that represent motor preparation or motor planning. The frontal eye field and supplementary eye field, located in the frontal cortex, are involved in the planning and preparation of saccadic eye movements and smooth-pursuit eye movements^42,43^. It is possible that observations of the EEG responses, especially in the frontal regions, could reflect these motor preparation signals (Fig. 4C). In the manual response condition, participants were not required to move their eyes to report their decisions; therefore, the movement preparation signal could be hand or arm-specific. The signal picked up with EEG electrodes might not be strong enough to modulate sensory motion representation. In the manual response condition, the neural direction discrimination lagged behind the involuntary eye drifts that were induced by the directional motion of the random dot patch (Fig. 5). This suggests that the EEG representation of sensory motion was weak when voluntary oculomotor behaviors were not planned, providing implications for the choice of motor behavior in the report of perceptual decision-making or detection when EEG activity is measured for the underlying neural mechanisms.

### Issues of artifactual influence of eye movements on the EEG activity

We found faster and stronger neural direction discrimination when oculomotor behavior was used as the motor action. It could be that this is simply due to eye movements or muscle artifacts. However, we argue against this possibility—we measured eye movement with sufficient spatial and temporal resolution (maximum spatial resolution of 0.01 deg and 1 kHz of temporal resolution) and compared these with EEG activities. First, significant neural direction discrimination occurred before any significant eye movements. When measuring eye velocity (which is more sensitive than eye position), we found that significant direction discrimination occurred at 114 ms after the motion target onset, and the start of the significant behavioral direction discrimination was almost identical across all three effector behavior conditions (Fig. 4A). When we measured behavioral latency, we did so through statistical methods for the increase in eye speed in the pursuit and saccade conditions and through button press timing in the manual response condition. (see Methods and Fig. 1B). We found that behavioral latency lagged behind the behavioral direction discrimination measured from eye drifts (average latencies were 196.33 ± 5.6, 158 ± 4.75, and 478.26 ± 16.74 ms for saccade, pursuit, and manual response, respectively).

Passive eye drifts induced by the motion stimulus would have the same neural origin as the ocular following^44,45^. Even if similar eye movements occurred during the first 125 ms after target onset, the neural responses were quite different, especially when the manual response and saccadic eye movement conditions were compared. Stronger and faster neural direction discrimination observed in saccadic eye movement conditions was not a passive outcome of eye movements or motor artifacts. Additionally, the cross-temporal analysis (Fig. 5) argues against the involvement of eye movement in neural direction discrimination. Eye drifts and their sensitivities to motion direction changes (that occurred roughly 114 ms after the target motion onset) were correlated with neural changes. However, in saccades and pursuit, neural direction discriminability that occurred in advance was correlated with ocular following or initiation of smooth pursuit, suggesting that these initial neural discriminations might influence behavioral direction discrimination. In the saccade condition, these initial neural responses were correlated with later saccadic eye movements and corrective saccades at the end of the open loop in the smooth pursuit eye movement condition. However, in the manual response condition, most of the significant neural direction discrimination lagged behind the behavioral discrimination evoked by eye drift. Finally, when a smooth pursuit eye movement was the effector behavior, active initiation of eye movement was performed approximately 158 ms after the motion onset (slower than a nominal pursuit latency of 100 ms because we used a lower contrast motion stimulus as a pursuit target). Therefore, behavioral latency, measured using the traditional method (Fig. 1B), was the shortest, and behavioral direction discrimination was the fastest and strongest among the three conditions. However, neural direction discrimination was weaker and slower in the smooth pursuit condition than in the saccade condition. If the observed neural direction discrimination was mainly due to eye movement artifacts, the effect would be strongest in the smooth pursuit condition. Thus, we argue that better neural direction discrimination in oculomotor behavior was mainly due to the effector-specific motor planning or preparation signal that strongly modulates sensory information, especially in saccade conditions. The EEG recordings readily picked up this cortical signal.

### The effect of motion direction prediction

A peculiar aspect of our results is that the neural direction discrimination occurred too early when the saccade was used as the effector behavior (Fig. 4B and C), when considering the time it takes for visual information to reach the visual cortical regions^46,47^. We think this might be due to participants being able to predict the direction of the upcoming motion. This may have been boosted by our experimental design; we allowed the participants to anticipate the target direction at least with a probability of ¼ by randomly shuffling between four directions (0, 315, 270, and 180). A set of four trials were composed of a random order of these four directions, therefore, participants may anticipate the upcoming motion direction and this expectation might hasten neural direction discrimination. To test whether anticipation of future motion direction has any effect on this, we performed an additional analysis using eye drifts during the fixation period as a proxy of motion direction anticipation (see Methods section for details). Under the assumption that motion anticipation would appear in the direction of the eye drifts^48^, we classified these based on their correspondence with the upcoming motion direction. We then sorted trials by this criterion and divided them according to whether the direction components of the drift matched with the upcoming motion direction (matching trials vs. non-matching trials). For all effector conditions, the matching trials showed faster neural direction discrimination than the non-matching trials. This suggests that motion direction expectations contributed to faster neural direction discrimination (Supplementary Fig. S5).

## Conclusions

Systematic investigation of the EEG representation of sensory motion under different effector behavior conditions revealed different degrees of neural sensitivity to motion direction changes. This suggests that using different motor effectors for the same scientific question could result in a completely different answer, at least in studies measuring cortical neural activity. The functional meaning and role of amplified neural motion direction discrimination need to be investigated in future studies to understand the interaction between sensory representation and movement-related planning signals.

## Supplementary Information

Supplementary Information.

## Data Availability

The data supporting the findings of this study are available from the corresponding author upon reasonable request.
