# Supplementary material for: Motor-effector dependent modulation of sensory-motor processes identified by the multivariate pattern analysis of EEG activity
Source: Sci Rep. 2023 Feb 23;13:3161. doi: 10.1038/s41598-023-30324-5 (PMC9950042; doi:10.1038/s41598-023-30324-5)
Supplement: Supplementary file 1 — Supplementary Information. [file 41598_2023_30324_MOESM1_ESM.docx]

Motor-effector dependent modulation of sensory-motor processes identified by the multivariate pattern analysis of EEG activity

Kahyun Choi^1,2+^, Sanghum Woo^1,2,3+^, and Joonyeol Lee^1,2,3*^

^1^Center for Neuroscience Imaging Research, Institute for Basic Science (IBS), Suwon, 16419, Republic of Korea

^2^Department of Biomedical Engineering Sungkyunkwan University, Suwon, 16419, Republic of Korea

^3^Department of Intelligent Precision Healthcare Convergence, Sungkyunkwan University, Suwon, 16419, Republic of Korea

**^+^**These authorscontributed equally to this work.

^*^Proofs and correspondence to:

Joonyeol Lee
Department of Biomedical Engineering
Sungkyunkwan University
2066 Seobu-ro, Jangan-gu, Suwon-si
Gyeonggi-do, 16419, Republic of Korea
Tel: +82 (31) 299-4359
Email: joonyeol@g.skku.edu

**Supplementary Figures**

**
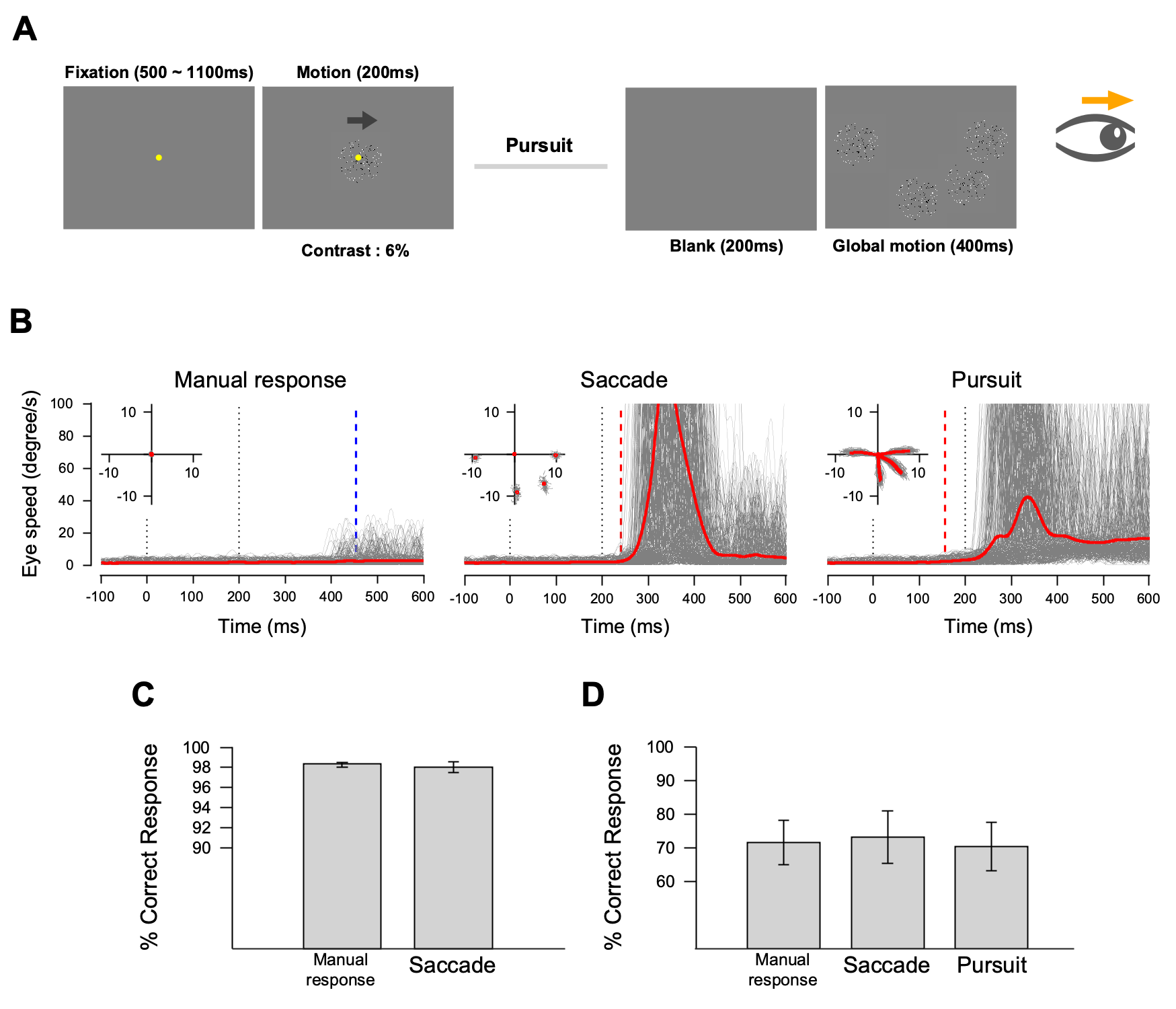
**

**Supplementary Figure S1. An experimental design of the modified pursuit task and behavioral results. (A)** The experimental design of the modified pursuit task. The luminance contrast of the motion stimulus is reduced to 6%. After the 200 ms of motion duration, we introduce another 200 ms duration of blank stimulus to evaluate the pursuit initiation induced by the motion stimulus. After the blank period, four patches appeared and moved to the four directions (0°, 180°, 270°, and 315°) with the 16°/s speed for 400 ms. **(B)** Eye speeds of a representative participant S1 for the main task with a manual response, saccade, and pursuit as the effector behaviors. The inset figures in each subplot show the participant’s individual and average eye positions in each direction. Gray lines show eye speeds from individual trials, and solid redlines show average eye speeds across trials. The blue dashed line in the left column shows the average reaction time measured from the button press, red dashed lines in the middle and right columns show the average reaction times measured from eye movements. The vertical black dotted lines indicate the start and the end of the motion period. **(C)** The percentage of correctly reported motion directions in the main experiment. The luminance contrast of the sensory stimulus was too high (25%) for evaluating the functional relevance of effector behavior types on perceptual performance. **(D)** Behavioral performances on different effector behaviors when the direction discrimination was challenging. The luminance contrast of the sensory stimulus was low enough (6%) to induce failures in motion discrimination. Error bars indicate the standard error of the mean.


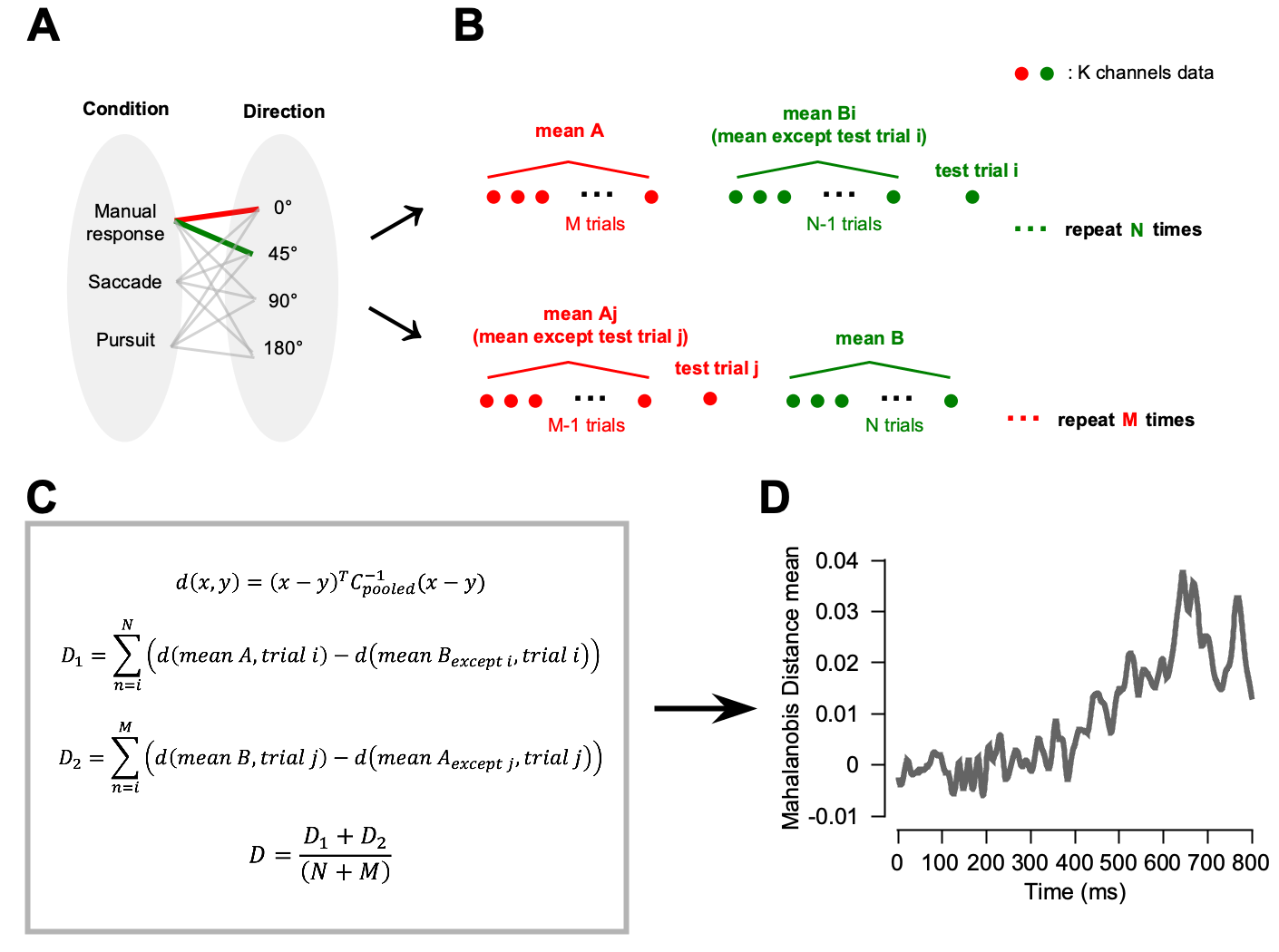


**Supplementary Figure S2. Schematic overview of Mahalanobis distance calculation. (A)** We estimated dissimilarity between two different sensory stimuli or effector behavior conditions using Mahalanobis distance. **(B) The** leave-one-trial-out procedure was used to prevent over-fitting. **(C)** Mathematical formulae used for calculating Mahalanobis distance and the dissimilarity calculation process. The dissimilarity is quantified by calculating D1 or D2 based on which group the test trials belong to and averaging them across trials. **(D)** The dissimilarity is estimated at each time point and plotted as a function of time.

**
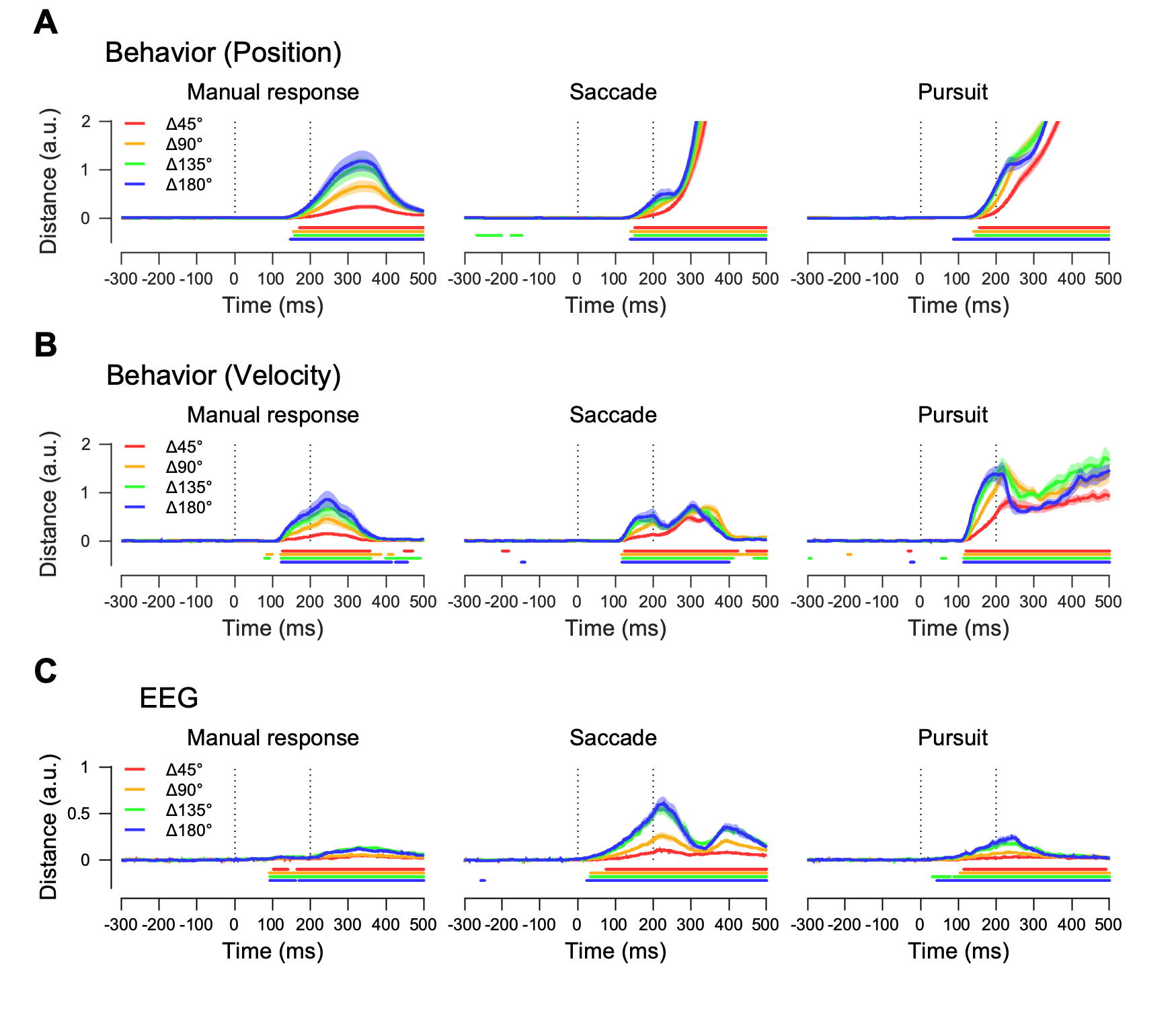
Supplementary Figure S3. Direction dissimilarity measured from the Mahalanobis distance in each direction difference condition. (A)** Eye position dissimilarities, **(B)** Eye velocity dissimilarities, and **(C)** EEG dissimilarities across motion direction differences in each motor-effector condition. Colored lines and shaded areas show the means and standard errors across participants in each direction difference condition, and vertical dotted lines indicate the motion period. Colored lines under the plot show the range where the Mahalanobis distances are significantly different from the baseline (two-sided cluster-based permutation test, *p*< 0.01).

**
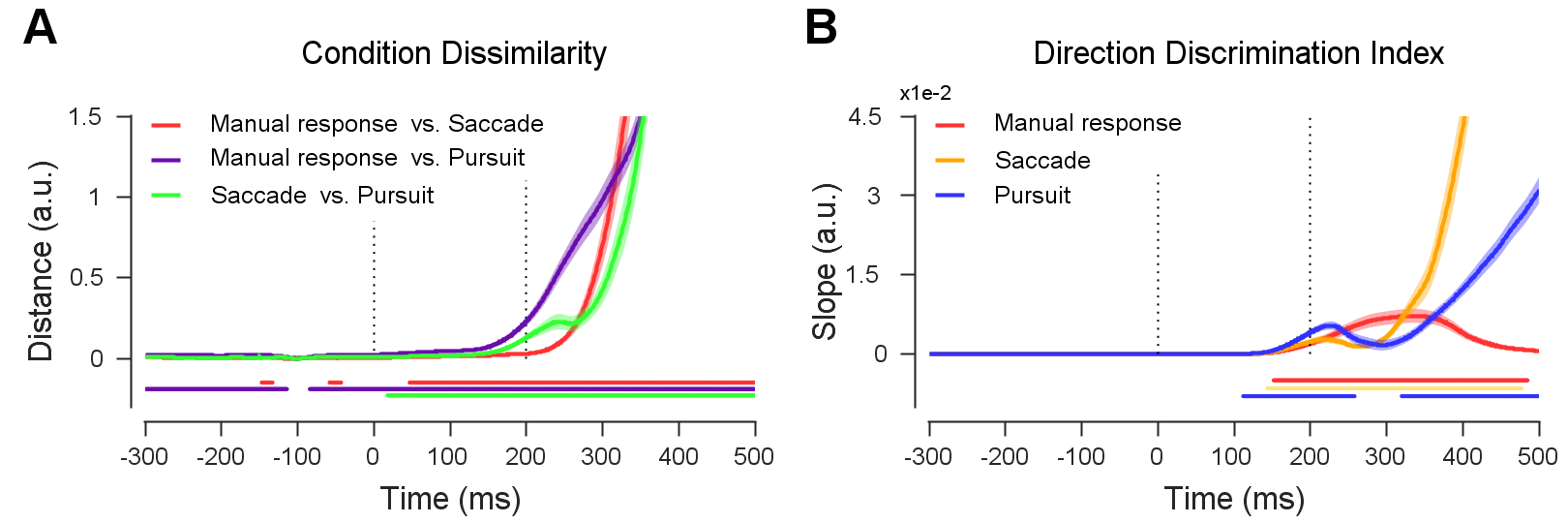
Supplementary Figure S4. Direction discrimination and dissimilarities across motor-effector conditions estimated from eye positions. (A)** Eye position dissimilarities across motor-effector conditions. **(B)** Direction discrimination index estimated from eye position dissimilarities. Mean and standard errors across participants are represented by colored lines and shaded areas, respectively. The motion period is marked with vertical dotted lines. Significant differences from the baseline are marked as color lines under the plot (two-sided cluster-based permutation test, *p*< 0.01).

**
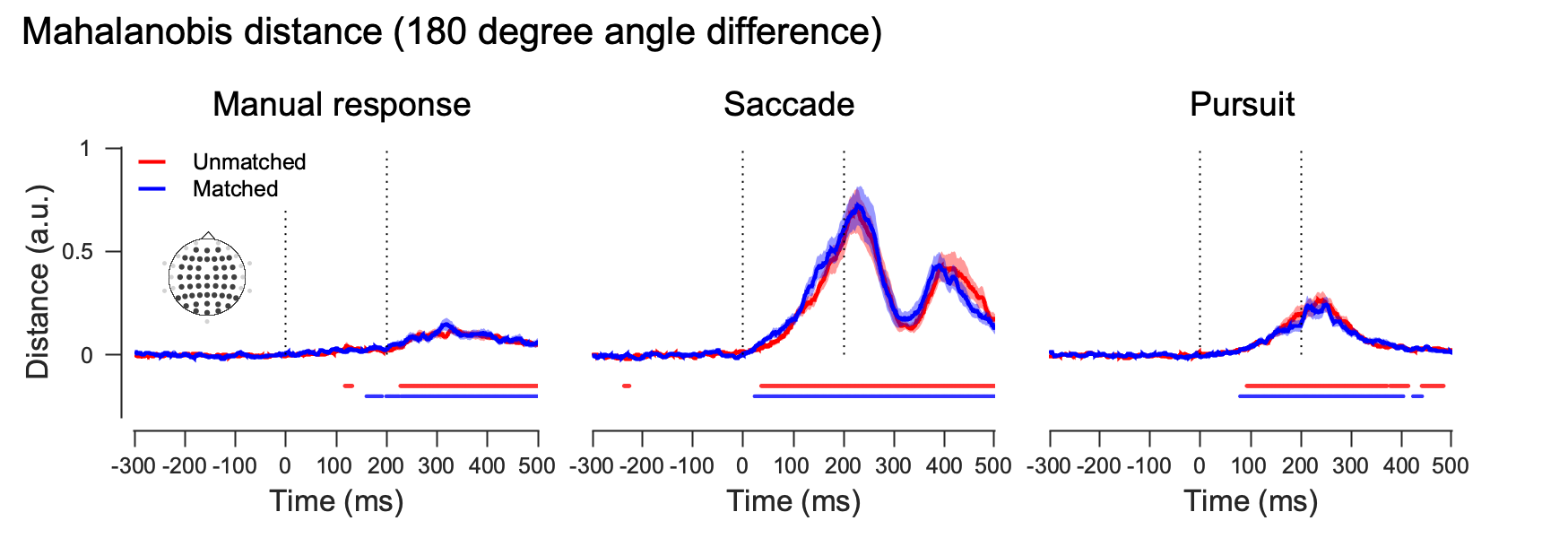
Supplementary Figure S5. The effect of direction anticipation on neural direction discriminations.** Mahalanobis distances of EEG activity pattern comparing the angular difference of 180°. In each effector behavior condition, we sort trials by how much the spontaneous eye movements show the directionalities that match with the upcoming target motion direction. Means and standard error across participants are represented by the lines and shaded areas, and the motion period is marked with vertical dotted lines. Colored lines under the plot indicate significant time duration where the Mahalanobis distances are significantly different from the baseline (two-sided cluster-based permutation test, *p*< 0.01). Significant direction discrimination occurs faster in direction-matched trials than in direction-unmatched trials.
